# The *Escherichia coli* PeptideAtlas Build: Characterizing the Observed *Escherichia coli* Pan-Proteome and Its Post-Translational Modifications

**DOI:** 10.1021/acs.jproteome.5c00902

**Published:** 2026-01-22

**Authors:** Caroline Jachmann, Zhi Sun, Kevin Velghe, Florence Arsène-Ploetze, Aurélie Hirschler, Jasper Zuallaert, Christine Carapito, Robbin Bouwmeester, Kay Nieselt, Eric W. Deutsch, Lennart Martens, Ralf Gabriels, Tim Van Den Bossche

**Affiliations:** † 360916 CompOmics, VIB Center for Medical Biotechnology, VIB, 9052 Ghent, Belgium; ‡ Department of Biomolecular Medicine, Faculty of Medicine and Health Sciences, Ghent University, 9052 Ghent, Belgium; § Institute for Systems Biology, Seattle, Washington 98109, United States; ∥ Institut de Biologie Moléculaire des Plantes, CNRS, 27083 Université de Strasbourg, 67000 Strasbourg, France; ⊥ BioOrganic Mass Spectrometry Laboratory (LSMBO), IPHC UMR7178, CNRS, Université de Strasbourg, 67200 Strasbourg, France; # French Proteomics Infrastructure ProFI, UAR2048, 31077 Toulouse, France; ∇ Institute for Bioinformatics and Medical Informatics, 9188 University of Tübingen, 72076 Tübingen, Germany

**Keywords:** reprocessing, PeptideAtlas, open search, Escherichia coli, modifications, proteomics

## Abstract

*Escherichia coli* is a widely used model organism in molecular biology. Despite its pivotal role, a comprehensive proteome resource covering the *E. coli* pan-proteome and its post-translational modifications (PTMs) has been lacking. Here we present the *E. coli* PeptideAtlas build, the first comprehensive pan-proteome analysis of *E. coli*, generated from 40 high-quality public and in-house data sets spanning a broad diversity of strains, sample types, and experimental conditions, and comprising over 73 million MS/MS spectra. All data sets were reprocessed using both a closed search (Trans-Proteomic Pipeline using MSFragger) and an open search (ionbot). The *E. coli* PeptideAtlas build provides evidence for 4755 proteins, including 1376 previously lacking protein-level support in UniProtKB. The resource offers protein coverage, modification sites, raw spectra with matched peptides, and manually annotated metadata for the *E. coli* pan-proteome. PTM profiling identified over 10,000 modification sites, including phosphorylation (3806), acetylation (754), methylation (730), glutathionylation (352) and phosphoribosylation (226). Analysis of the glutathionylation sites revealed potential links to metal binding regulation. We also detected proteins likely stemming from phages, underscoring the value of pan-proteomic approaches for studying host-phage interactions. All identifications are publicly accessible and traceable through the PeptideAtlas interface. We expect that the *E. coli* PeptideAtlas build will provide a useful resource for the community, which supports, for example, targeted MS experiment design, PTM enrichment method development, and strain typing. It allows straightforward lookups of protein and peptide identifications and facilitates comparative proteomic analyses by enabling the assessment of protein presence and variability across different *E. coli* strains. The build is available at https://peptideatlas.org/builds/ecoli/.

## Graphical abstract

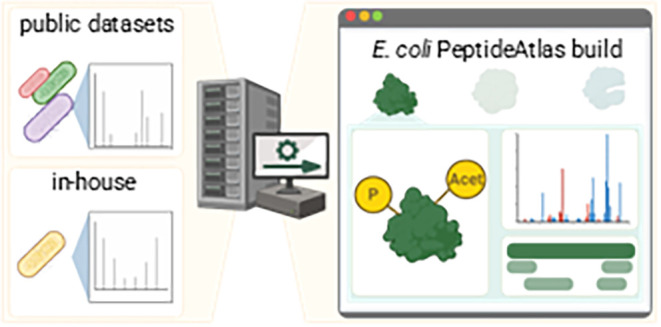

## Introduction

*Escherichia coli* (*E. coli*) is a cornerstone in biological research, serving as a key model organism in various fields, including biotechnology and biopharmaceutical research.^1^ Its extensive biological diversity ranges from probiotic strains beneficial to humans to pathogenic strains associated with diseases such as diarrhea, urinary tract infections, and meningitis.^2^ The species exhibits vast genomic diversity, with a pan-genome encompassing up to 18,000 genes,^3^ of which only a small portion is shared across all strains. This genetic variability is driven by the species’ high genomic plasticity,^1^ facilitating rapid adaptation via horizontal gene transfer.

Despite the wealth of genomic information, proteomics data for *E. coli* remain fragmented. Most studies focus on a few K-12 derivatives, using the MG1655 reference proteome. The largest *E. coli* proteome profiling studies to date have identified up to 3300 proteins,^4^ with quantification data available for 2300 proteins.^5^ These studies have examined the *E. coli* proteome under diverse conditions, including different growth media and stages, which is essential for detecting condition-specific proteins. Smaller scale projects have also explored specific conditions like antibiotic stress,^6^ high temperature,^7^ and even spaceflight.^8^

Thanks to the adoption of open data standards in proteomics, these and many other *E. coli* data sets are now available in public repositories like ProteomeXchange.^9^ While these data are valuable, they often lack reprocessing or integration into comprehensive resources. Indeed, while *E. coli* is the most represented prokaryote in ProteomeXchange, no unified reprocessing has been conducted until now. Moreover, existing resources such as PeptideAtlas provide valuable tools for building proteome profiles but have yet to offer a comprehensive *E. coli* resource, especially one that captures the protein and post-translational modification (PTM) landscape in multiple strains and conditions. And perhaps most importantly, many protein entries in UniProtKB^10^ still lack experimental evidence at the protein level, limiting their utility in functional or comparative studies.

In this study, we present the first systematic reprocessing effort to characterize the expressed *E. coli* pan-proteome and its modifications. By integrating public and in-house LC-MS data with the Trans-Proteomics Pipeline^11^ (TPP) and ionbot,^12^ an open-modification search engine, we created the *E. coli* PeptideAtlas build. This resource includes both core and strain-specific proteins, providing MS-based evidence for protein entries in UniProtKB previously annotated as “predicted” or lacking experimental validation. We also identified a wide range of PTM types and modification sites, contributing to their functional annotation. The PeptideAtlas build is publicly accessible and supports various applications, including targeted MS assay development, method development for PTM enrichment, and comparative proteomic analyses across different *E. coli* strains.

## Methods

### Data Collection

LC-MS/MS projects available through ProteomeXchange were filtered for (1) only containing *E. coli*, and (2) data acquisition performed on Orbitrap instruments (Q Exactive, LTQ Orbitrap), with the exception of PXD020785, a large spectral library assay acquired on TripleTOF instruments. The resulting projects were then manually filtered for projects using HCD fragmentation and trypsin or a combination of trypsin and LysC as a cleavage agent, ensuring compatibility with ionbot (again, with the exception of PXD020785, which was only analyzed with TPP). Metaproteomics and cross-linking experiments were excluded. These publically available data were supplemented with in-house acquired data for *E. coli* strain W3110 ^13^ under six different conditions (see next section for details).

In total, 40 projects^4–6,8,14–45^ were selected for the build. Employed methodologies include solubility-based fractionation (1 project), pH-based fractionation (2 projects), and pre-enrichment for certain modifications and cellular components, such as phosphorylated peptides, lactyllysine-modified peptides, outer membrane proteins, and membrane vesicles. An overview of the 40 included projects, as well as more extensive descriptions including e.g., utilized media and growth stages, is given in Supporting Table S1.

### Sample Preparation

In addition to public data, we also included in-house data acquired on different growth media and growth stages. First, 50 mL of M9 + glucose (2 g/L) were inoculated from a 5 mL culture of *E. coli* strain W3110 grown overnight in LB and incubated at 37 °C with agitation. On the second day, this 50 mL culture was used to inoculate two different 100 mL media (LB (LB Lennox Agar Broth, Euromedex, Souffelweyersheim, France) or M9 (M9CA Medium, Euromedex, Souffelweyersheim, France) + glucose (2 g/L)) to obtain an initial OD of 0.01 (in LB) or 0.02 (M9 + glucose), with four replicates each. The cells were cultured at 37 °C with agitation. After 8 h, the 100 mL cultures of M9 + glucose reached an OD of approximately 0.1. 2 × 50 mL of each of these cultures were centrifuged for 10 min at 5000 rpm. One of the two pellets was resuspended in 50 mL of M9 + glucose (2 g/L) and the other pellet was resuspended in 50 mL + acetate (2 g/L) and incubated at 37 °C with agitation. Under each culture condition, cells were sampled at two growth stages (exponential growth stage, OD ≈ 0.6, and late stationary phase, 72 h).

Proteins were extracted from frozen cell pellets by resuspension in Laemmli like buffer, sonication with Bioruptor Pico (10 times, 30s on/off) and centrifugation (5 min, 10,000 g). Protein concentration was measured using the Pierce 660 nm Protein Assay kit. In-gel protein digestion was performed according to standard protocols. Briefly, the samples were heated for 5 min at 95 °C, loaded on in-house stacking gels (40 μg), and the gels were run at 40 V until proteins were migrated around 1 cm into the gel. Gels were fixated for 15 min, stained with Silver Blue for 1 h, and the bands were excised and transferred to 96-well plate. Proteins were decolored 4x with ACN/NH4HCO3, dehydrated with 100 μL ACN for 5 min, reduced with 60 μL of 10 mM DTT for 30 min at 60 °C and 30 min at room temperature. Proteins were alkylated with 60 μL of 55 mM IAA for 20 min in the dark, washed 4× with 80 μL NH4HCO3, 80 μL of ACN for 5 min each, and dried with 80 μL of ACN twice for 5 min. Then, proteins were digested overnight at 37 °C using modified porcine trypsin with a final trypsin/protein ratio of 1/100 (Promega, Madison). Peptides were extracted by adding 100 μL ACN (60%) for 1 h under gentle shaking, and 60 μL ACN (100%) for 10 min without. Peptides were dried in a vacuum concentrator and dissolved in 100 μL H20, 0.1% FA, 2% ACN.

### LC-MS/MS Acquisition

Data-dependent acquisition was done on a nanoAcquity Ultra Performance LC device (Waters Corporation, Milford, MA) coupled to a quadrupole-Orbitrap mass spectrometer (Q-Exactive HF-X, Thermo Fisher Scientific, Waltham, MA). A 58 min stepwise gradient was applied (0.1% FA in water (solvent A) and 0.1% FA in ACN (solvent B), 1–35%B). MS1 spectra were acquired at a resolution of 60,000, MS2 spectra at 15,000. Peptide fragmentation was performed using HCD (NCE: 27%). Dynamic exclusion time was set to 30s, AGC target was set to 3 × 10^6^, the mass range was set to 300–1800 *m*/*z*, and the maximum injection time was set to 50 ms. The metadata was annotated with lesSDRF.^46^ Raw files and associated SDRF^47^-formatted metadata were uploaded to PRIDE and are available under the identifier PXD058808.

### Metadata Collection and Annotation

Information about all publicly available samples and experiments were also annotated in SDRF-format with lesSDRF. These machine-readable files include information necessary for reprocessing (mass tolerances, labeling, enrichment, among others) and for downstream analyses and interpretation (such as growth media, strain, treatment) down to the level of each MS run. Where necessary, original authors were contacted for more information. These metadata are available in Supporting Table S1.

### Construction of the Proteome Search Space

To capture sequence variation while limiting the search space and reducing false peptide-spectrum matches, an *E. coli* pan-proteome search database was constructed by combining multiple strain proteomes. Representative strain proteomes were sourced from UniProtKB whenever available. For strains without publicly available proteomes, closely related strains were identified through literature searches. Seven strains had available proteomes: MG1655, DH5α, BW25113, MC4100, ATCC 25922, BL21, and W3110. For W0153 and BW25993, no direct proteomes were found, but BW25993 was closely related to BW25113 (already included), and for W0153, the proteome of its parent strain AB1157 was included. No proteomes were available for AE17, DSM 105380, BA102, and AG1 (ME5305). For the clinical samples in PXD050358, the whole-genome sequencing-based proteomes provided in the original study were included.

Swiss-Prot bacteriophage proteins were also included to account for phage-derived proteins that may be present in *E. coli* strains. Additionally, known contaminants (excluding *E. coli* proteins) were incorporated from Frankenfield et al.^48^

The final FASTA search database was constructed by concatenating all selected proteomes. After removing duplicate sequences, this yielded a total of 28,580 target sequences. To ensure robust false discovery rate (FDR) control, an equal number of decoy sequences (28,580) was generated by shuffling target sequences while preserving K and R positions. A complete list of included proteomes, along with their UniProt accession numbers and download dates, is provided in Supporting Table S1, proteome sizes are shown in Supporting Figure S2. The FASTA database itself (with and without decoy protein sequences added) is available for download in the GitHub repository (https://github.com/CompOmics/ecoli-peptideatlas-manuscript/).

### LC-MS/MS Data Processing

The public and in-house data sets were processed both in a closed search approach (TPP, with MSFragger^49^ as the search engine), and in an open search approach (ionbot^12^).

For TPP reprocessing, the raw mass spectrum files were downloaded from the ProteomeXchange repository and converted into mzML format.^50^ The conversion process utilized ThermoRawFileParser^51^ version 1.4.3 for Thermo files, while SCIEX MS Data Converter 1.3.1 ^52^ was used for. wiff files from SCIEX instruments. Data analysis was performed using TPP with MSFragger as the search engine, leveraging parameters sourced from the SDRF files associated with each data set, where specific parameters were not detailed, default settings were applied. The analysis included a fixed modification of carbamidomethylated cysteine, variable modifications include oxidized methionine, protein N-terminal acetylation, pyroglutamic acid formation from glutamine and for cyclization of N-terminal *S*-carbamoylmethyl-cysteine, pyroglutamic acid formation from glutamic acid, and deamidation. Exact mass shifts for these modifications are listed in Supporting Table S1. In cases where experiments involved isobaric or metabolic labeling strategies, labeling-specific modifications were added accordingly. Precursor mass tolerance was set to 10 ppm. Fragment mass tolerance was set to 20 ppm, except for PXD030345 and PXD030346, where it was set to 0.6 Da (due to fragment ion analysis in the ion trap). For PXD020785, both precursor and fragment mass tolerances were set to 50 ppm. The minimum peptide length was set to seven amino acids. Up to two missed cleavages were allowed, and semitryptic cleavage was used. The False Localization Rate of phosphorylation site assignments were estimated by including (chemically impossible) alanine phosphorylations in the search space as described by Ramsbottom et al.^53^

Additionally, an open search was performed with ionbot v0.11.4. The raw files were converted to. mgf format using ThermoRawFileParser version 1.4.4. ionbot searches for peptides with maximum two variable modifications plus maximum one unexpected modification. Therefore, we specified cysteine carbamidomethylation and methionine oxidation as variable modifications to allow for the identification of peptides carrying unexpected modifications on top of those expected modifications. Carbamidomethylation was not applied as a fixed modification to allow for incomplete modification and the possibility of cysteines being modified by other modifications, as cysteine residues are highly reactive and can undergo a variety of modifications under different experimental conditions. This flexibility ensures that the search accounts for a broader range of potential peptide variations. Additionally, fixed and variable modifications were set accordingly for projects using isobaric labels and PTM enrichment. Semitryptic peptides were included, and a maximum number of two missed cleavages were allowed. Precursor and fragment mass tolerances were left at the default values (20 ppm), and rescoring was performed with Percolator.^54^ A very strict PSM FDR threshold of 0.05% per run was applied to only include highly confident identifications. Additionally, modifications were relocalized with pyAscore,^55,56^ and only PSMs with high confidence (Ascore >20) were integrated into the PeptideAtlas build.

### Creating the PeptideAtlas Build

The results from the closed and open search were combined into one PeptideAtlas build available under https://peptideatlas.org/builds/ecoli/.

In case of conflicting peptidoform assignments between ionbot and TPP, the closed search result was used. After merging the PSMs, MAYU^57^ was used to ensure a global protein FDR below 1%. PeptideProphet^58^ was employed to evaluate the confidence of PSM identifications, providing probabilities to each identified peptide. Spectral libraries were created with SpectraST.^59^

When working with multiple proteomes, PeptideAtlas employs a designated *core proteome* that takes precedence during protein inference. This should not be confused with the core proteome in the context of pan-proteomics, which refers to the set of proteins shared across all strains. For this, we chose UP00000625 (K-12 MG1655), as it is the reference proteome for *E. coli* in UniProt and is assumed to be close to completely annotated.^60^

Depending on their evidence, protein identifications are categorized into one of ten tiers in PeptideAtlas builds. For further analysis in this paper, we use a simplified version of the PeptideAtlas tier system similar to a previous PeptideAtlas build^61^ (Table 1): The “representative” tier was removed as there were no proteins in this tier in this build. Identical proteins are assigned to the tier of their (identical) partner but are not counted again for summary statistics. Tiers 5, 6, and 8 are combined into one Tier (“uncertain”).

**Table 1:** Simplified Confidence Tiers for This Study

tier	contains
(1) canonical	high confidence proteoforms that can be completely distinguished from other proteoforms and/or are in the K-12 MG1655 reference proteome
(2) indistinguishable from canonicals	high confidence proteoforms that do not have a uniquely mapping peptide that distinguishes it from a canonical proteoform
(3) marginally distinguished from canonicals	high confidence proteoforms that have a uniquely mapping peptide that distinguishes it from a canonical proteoform
(4) uncertain (combines Tier 5, 6, 8, and proteins indistinguishable from noncanonical proteins)	proteoforms that cannot be distinguished from other proteoforms and/or proteoforms with low confidence
(5) not observed	proteoforms without considered peptide evidence

### Terminology

The search proteome includes multiple strain proteomes and, consequently, contains a substantial proportion of proteins with highly similar sequences. We will therefore refer to FASTA entries as proteoforms in this paper. Clustering of proteoforms gives rise to homology clusters, representing proteoforms of the same protein. There is no universally agreed on similarity threshold to choose for protein clustering, and for this paper we will use 70% as default, as done in Broadbent et al.^62^ Homology clusters with at least one proteoform in every included strain are taken as “core proteins”; clusters with at least one proteoform in at least two, but not all strains are referred to as “accessory proteins”, and proteins only found in one strain proteome are called “orphan proteins”.

### Proteome Characterization and PTM Analysis

Protein subcellular localizations were predicted with PSORTb 3.0,^63^ isoelectric points and weights were predicted and calculated, respectively, with Pyteomics.^64^ Protein clustering was done with CD-HIT^65^ v4.8.1–2019, the word size was set depending on the similarity threshold as recommended in the manual. Proteins identified with high confidence (termed canonical proteins) in the build were aligned to all UniProtKB *E. coli* proteins with known evidence (existence:1 and taxonomy_id:562, 4351 proteins as of 27.9.24) to evaluate how many proteins gained new experimental evidence. For this, any canonical proteins that did not match with a similarity of 70% or higher to the already observed proteins were considered new. To estimate proteome similarities, pairwise Jaccard similarities were calculated on tryptic digests. For the PTM site analyses, peptides carrying modifications were remapped to canonical proteins to filter out modification sites that are not uniquely mapping to one protein. Protein abundance was estimated by the normalized spectrum abundance factor (NSAF)^66^ calculated by counting the number of PSMs mapping uniquely to the protein and dividing it by the length of the protein. Additionally, the NSAF was normalized by the number of all PSMs found in the run. Batch effects in modification patterns were investigated using t-SNEs, which were calculated based on the observation counts for each PTM per run, i.e., each run is represented by a vector specifying how many times each PTM was found in that run. We employed ESMFold^67^ (esm.pretrained.esmfold_v1 model) for protein structure prediction on account of its computational efficiency.

## Results

### *E. coli* PeptideAtlas Build

To support high-confidence and strain-resolved *E. coli* proteomics, we constructed a comprehensive *E. coli* PeptideAtlas build by integrating publicly available LC-MS/MS data sets from ProteomeXchange with in-house generated data (Figure 1A). The selected 40 projects encompass a broad array of experimental conditions and analytical strategies, including diverse *E. coli* strains, antibiotic and environmental perturbations, sample preparation protocols, and enrichment methods designed to enhance the detection of low-abundance or modified peptides (Figure 1B). In total, over 73 million spectra were reanalyzed using a dual search strategy: a closed search with MSFragger via the Trans-Proteomic Pipeline (TPP) and an open search with ionbot (Figure 1C). These results were then combined under strict protein-level false discovery rate (FDR) control using MAYU.

**Figure 1:**
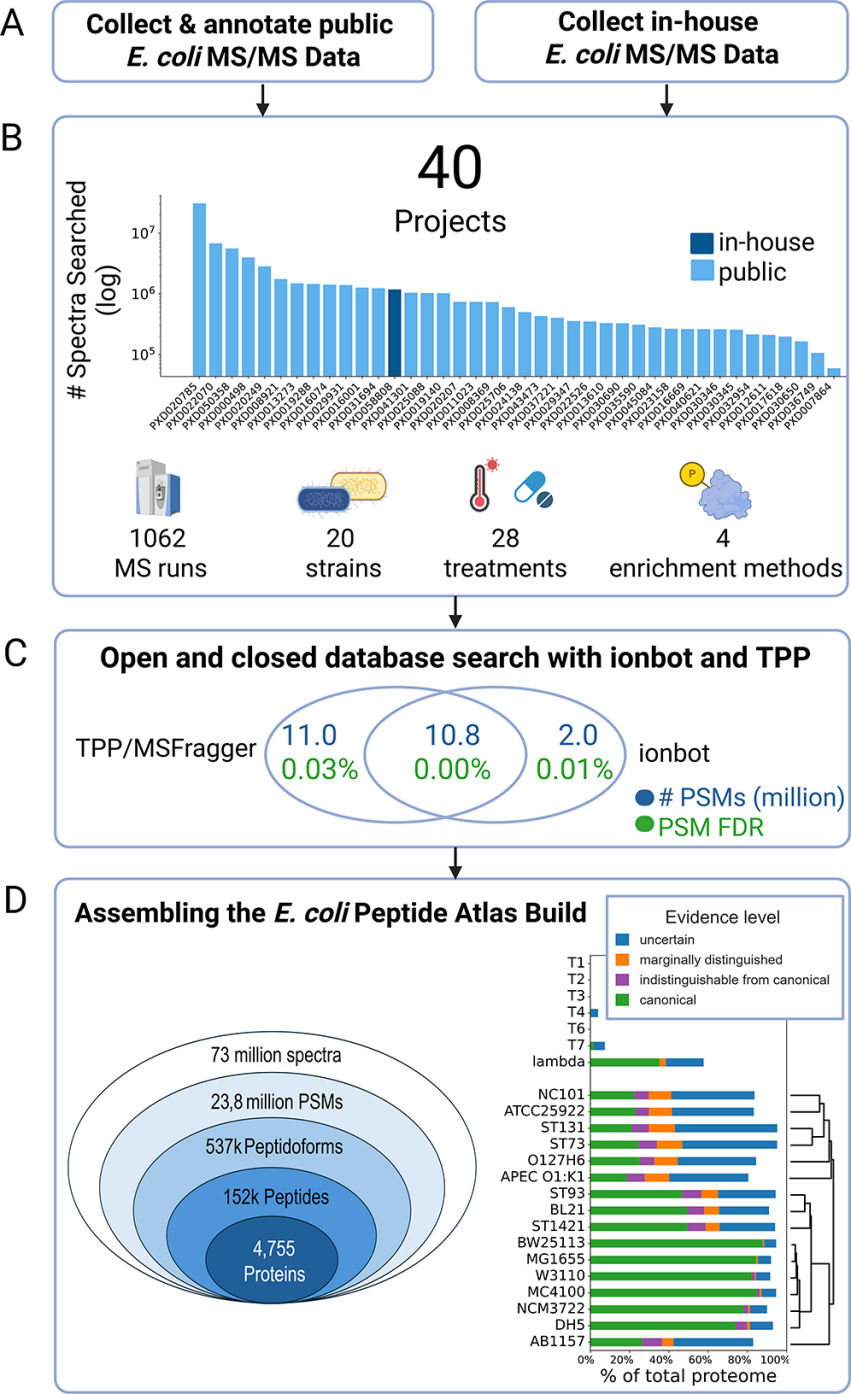
*E. coli* PeptideAtlas build. (A) *E. coli* PeptideAtlas build was created by combining public proteomics data from ProteomeXchange and LC-MS/MS data acquired in-house. (B) Selected experiments cover a large variety of strains, treatments, sample preparation and enrichment protocols. Spectral counts per experiment are shown in bar chart. (C) Spectra were reprocessed with closed MSFragger search via TPP and open search with ionbot, and results were combined under strict global protein FDR control with MAYU. (D) 537,885 distinct peptidoforms were identified, giving evidence to 4755 proteins in the highest confidence tier. Proteome coverages vary between 76% and 90% across strains including all confidence tiers. As we prioritized K-12 MG1655 proteins in case of indistinguishable proteoforms during protein inference, coverages for strains not derived from K-12 are generally lower. The tree next to the proteome coverage bar plot is based on the distance of tryptic digests between the proteomes.

This yielded over 537,000 distinct peptidoforms and provided peptide-level evidence for 4,755 canonical proteins (Figure 1D). The coverage across reference proteomes varies between 76–90%, with a higher canonical coverage in K-12-derived strains due to prioritization of K-12 MG1655 proteins during inference in cases of shared peptide evidence. The hierarchical relationship between proteomes, based on in silico tryptic peptide overlap, underscores both their diversity and the value of incorporating multiple strain backgrounds (Figure 1D, right). This build not only provides a curated, high-resolution view of the *E. coli* proteome but also supports functional annotation and system-level exploration by linking identified peptides and proteins to experimental metadata and external databases.

#### Selected 40 Projects Cover a Large Variety of Experiment Types

The projects collected from public repositories or generated in-house showcase the diverse experimental approaches applied to *E. coli* research globally, encompassing a variety of enrichment and fractionation techniques tailored to enhance the detection of low-abundance peptidoforms and traditionally elusive proteins via LC-MS/MS (see Figure 1B).

These studies investigated proteome changes under numerous environmental conditions, including exposure to 21 different antibiotics and physical conditions like high temperature (42 °C), increased pressure, and spaceflight. Contributions have come from around the globe, with a significant number from Europe (14), followed by Asia (13), America (8), Oceania (2), and Africa (1). Labeling techniques employed in these studies include tandem mass tags (TMT) (4), stable isotope labeling by amino acids in cell culture (SILAC) (3), and isobaric tags for relative and absolute quantitation (iTRAQ) (1).

Both laboratory strains and clinical isolates are represented, with a predominance of *E. coli* K-12 derivatives like MG-1655 (9 projects) and W3110 (5 projects). Additional lab strains used include BLR­(DE3), HMS174­(DE3), BL21­(DE3), and ATCC 25922. Together, these strains represent different phylogenetic (A, B2–1, and B2–2) and pathogenic groups (AIEC, APEC, and ExPEC), with samples sourced from chickens, humans, and mice.

Some studies induced genetic modifications such as gene knockdowns (1), gene knockouts (7), and the introduction of plasmids (5). The proteins encoded by these artificially added genes are not included in this study. Further information on the included plasmids and gene edits is listed in Supporting Table S1.

To ease exploration of the results, each of the 40 projects were further divided into smaller experiments to account for different experimental setups such as growth media, fractions, strains, and treatments. All experiments are listed in Supporting Table S1.

#### Combining Closed and Open Search Methods Leads to High Identifications at Controlled FDR

The curated 40 projects sum to 73,008,556 spectra from 1062 MS runs. TPP and ionbot identified 23,823,673 (33% of all spectra) PSMs at a 1% global protein FDR, mapping to 537,885 distinct peptidoforms and 151,590 distinct peptides (Figure 1C,D). Around half of the PSMs (10.8 million) included in the build were identified in both the open and closed search, and TPP identified an additional 10.8 million PSMs. The open search identified an additional 2 million PSMs from spectra previously unassigned by TPP, 28.6% (576,444) of which contained modifications not considered in the closed search. For the 3,051,960 PSMs with conflicting peptidoform assignments between ionbot and TPP, the peptidoforms assigned by TPP in the closed search were added to the build.

Based on the collected peptide evidence, a total of 4755 proteins were identified with high confidence (Tier 1). All results are available online through the build page on the PeptideAtlas Web site, presenting protein overviews with coverages, and strain and experiment provenance information. It is also possible to trace evidence back to original spectra, and to inspect annotated spectra in a viewer. Moreover, cross-references to databases such as UniProtKB, KEGG,^68^ and NCBI^69^ enable users to access additional information on existing biological knowledge, including details on PTMs.

The spectrum matching rates differ between experiments (subsets of runs in a project with the same experimental setup), from lower than 1% of spectra identified in single bacterium runs, to 80% of spectra acquired in standard bulk setups. The median ID rate is 42.8%, with higher ID rates for runs on Q Exactive instruments, and lower rates on Orbitrap Fusion, Eclipse, and Velos (Supporting Figure S1).

#### PeptideAtlas Tier System Allows for Easy Analyses at Different Confidence Levels

Proteins identified in PeptideAtlas builds are assigned to tiers based on the observed peptide evidence. Similar to a previous study,^61^ we simplified it to four tiers (see Methods Section). 4755 proteins were assigned to the highest tier (“canonical”), meaning that they have at least two uniquely mapping peptides. Of these, 1246 are not part of the K-12 reference proteome, the most commonly used reference database for *E. coli* proteomics analyses. Peptides from 4074 additional proteins were observed, but they could only be distinguished from other peptidoforms based on one uniquely mapping peptide (“marginally distinguished”).

The relative coverage varies across the selected reference proteomes (Figure 1C), with K-12 derivatives having the least percentage of unobserved proteins (around 5%), and pathogenic strains like NC101 and APEC the most (around 20%). As the K-12 MG1655 proteome was selected as the core proteome for the build, the number of canonical proteins for K-12 derivatives is higher due to proteins from this proteome given preference in case of ambiguity.

#### Strong Experimental Evidence for 1376 Proteins Not Observed until Now

Of the 4,755 canonically identified protein isoforms in the *E. coli* PeptideAtlas build, 1376 had no prior protein-level evidence in UniProtKB, even when accounting for evidence linked to homologous entries. Among these, 785 proteins originate from the well-studied K-12 MG1655 reference proteome. Notably, this set includes 358 proteins annotated as uncharacterized and an additional 541 proteins with limited existing knowledge, reflected by UniProtKB annotation scores below 3. For these poorly annotated MG1655 proteins, the PeptideAtlas resource offers valuable new evidence by indicating the specific strains and experimental conditions under which peptide support was observed, thereby providing a foundation for refining functional annotations and guiding future experimental investigations.

#### Webpage Offers Extensive Information on PSM, Peptide, Protein, and Proteome Level

Upon accessing the build overview page, users are presented with summary statistics, including the total number of distinct peptides, post-translationally modified peptides, and protein identifications, alongside metrics such as the number of projects included and the search engine workflows applied. The interface allows intuitive navigation between the peptide, protein, and PSM levels, each of which provides layered insight into the *E. coli* proteome. On the protein level, users can explore proteins filtered by tiers, view supporting peptide evidence and from which experiments and strains they come from, determine the number and types of peptides matched, and assess evidence quality and coverage via visual tools such as protein coverage maps (Figure 2A). On the peptide level, the site offers overviews on peptide proteotypicity, its respective modification sites, and the number of PSMs supporting each peptidoform. Users can further interrogate PSMs, including raw spectrum annotations (Figure 2B) and quality metrics, aiding in the evaluation of peptide identification confidence. The interface also enables queries by protein accession and peptide sequence and facilitates downloading of filtered or full identification lists for downstream analyses. Modifications can further be inspected interactively with PTMVision,^70^ which has been extended to parse the Mass Modification Locations Table provided on the build page of each protein. PTMVision allows users to obtain an overview of PTMs on protein sequence and structure, and to inspect sites in more detail, e.g., with additional context from UniProtKB and residues in close contact to PTMs (Figure 2C,D).

**Figure 2:**
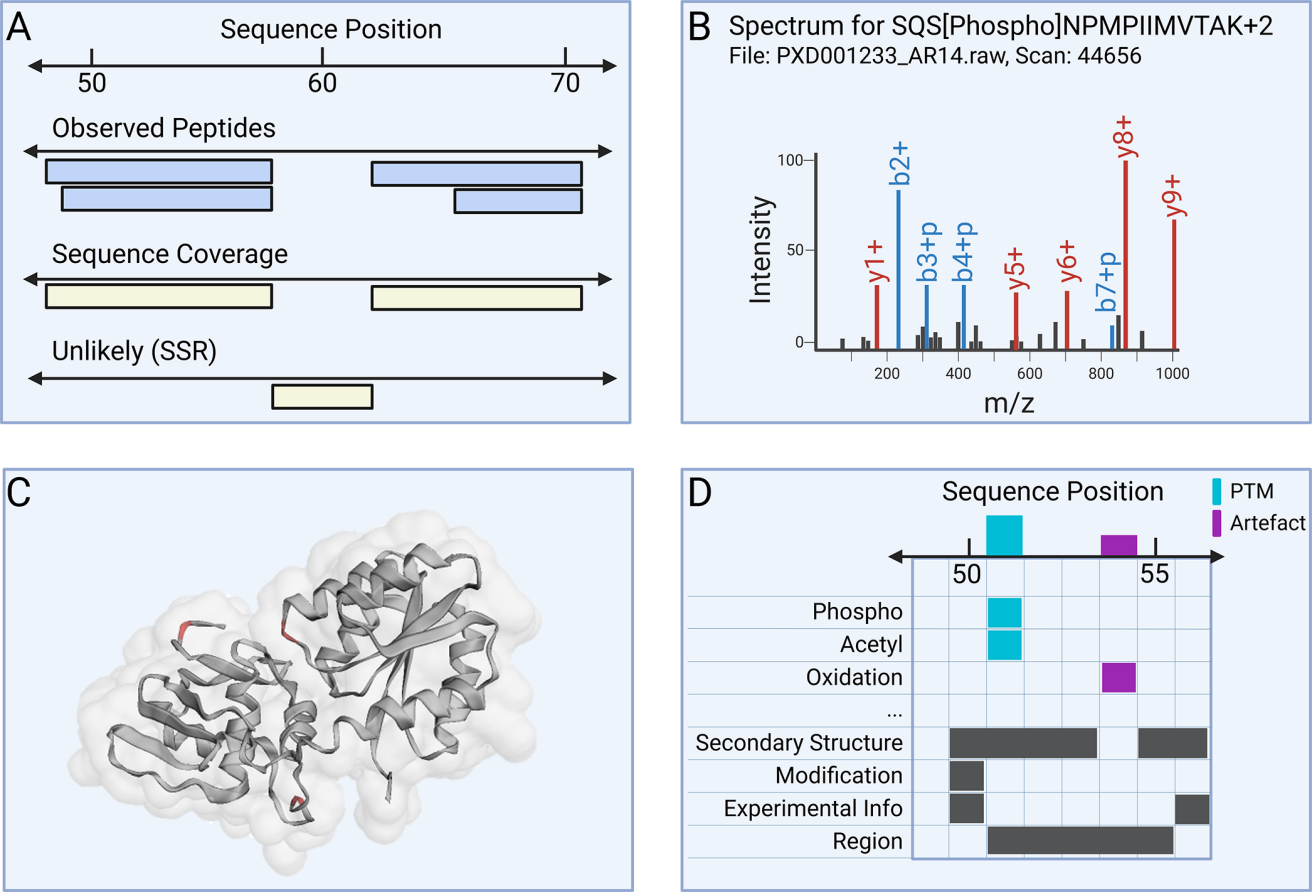
*E. coli* PeptideAtlas offers different ways to visually explore results. (A) Alignment of observed peptides along amino acid sequence, showing protein coverage with additional peptide information. (B) Peptide evidence can be traced back down to the original spectrum, which can then be inspected in an annotated view. (C, D) Modification site data from the build can be uploaded to PTMVision to inspect modification sites interactively in both 3D (C, phosphorylations highlighted) and 2D (D).

#### Bacteriophage Peptides were Detected in Multiple Projects

Despite strict efforts to maintain sterile conditions, bacteriophage contamination remains a major challenge in both industrial and laboratory settings, particularly during large-scale cultivation procedures.^71^ In addition, certain *E. coli* strains naturally harbor prophage sequences within their genomes; among the strains examined, only DH5α was reported to contain five prophage sequences.^72^ Peptides mapping uniquely to the phage proteins were detected in 17 out of 40 projects, with no observable preference for specific *E. coli* strains. Certain phages, such as T1, T2, T3, and T6, did not yield any detectable proteins at any confidence level. In contrast, T4 presented nine proteins with weak confidence (below Tier 1) and one with high confidence. For T7, three proteins were detected, including one canonical protein. Notably, 22 of 66 lambda phage proteins were identified with high confidence. An additional 11 proteins were identified with weak confidence, while two were marginally distinguished, and one protein remained indistinguishable from other phage proteins. Furthermore, peptide evidence for 16 of the 22 canonical lambda phage proteins was found in one specific data set (PXD041301, strain DSM 105380) that included samples enriched for outer membrane vesicles. This preparation method likely enhances the detection of phage proteins because outer membrane vesicles are a defense mechanism of Gram-negative bacteria and work as “decoys” that carry membrane receptors that bacteriophages bind to.^73^

### Predicted and the Observed *E. coli* Pan-Proteome

To gain a comprehensive view of the *E. coli* pan-proteome, we clustered proteins from selected reference proteomes using varying similarity thresholds, allowing us to classify protein families into core, accessory, and strain-specific orphan groups. This approach provides insight into the structural organization of the pan-proteome and its relevance across diverse *E. coli* strains. By linking these clusters to peptide identifications from the PeptideAtlas build, we further evaluated which parts of the proteome are readily detectable by mass spectrometry and which remain undetected. Our analysis demonstrates that many proteins go unobserved due to physicochemical properties that hinder their identification, such as small size, limited tryptic peptide yield, and membrane localization. Nonetheless, the use of diverse enrichment and fractionation strategies enabled the detection of a broad range of proteins, including those typically difficult to identify, and revealed strain-specific candidates with potential utility for typing and functional characterization.

#### Homology Clustering and Pan-Proteome Composition

Clustering the proteins included in the selected reference proteomes at varying sequence similarity thresholds revealed a clear and expected trend in the relationship between the number of homology clusters and their mean size (Figure 3A). As the similarity threshold decreased, the number of clusters diminished, while the mean cluster size increased, indicating greater aggregation of homologous sequences. At a 70% sequence similarity threshold, the pan-proteome, consisting of 27,806 distinct proteoforms, was divided into 8310 homology clusters, with an average of 3.3 proteoforms per cluster. This suggests that, on average, each *E. coli* protein has at least three distinct proteoforms; the distributions are shown in Supporting Figure S3. The homology clusters were further classified into core, accessory, and orphan categories (Figure 3B). Of these, 2871 clusters (35%) were present in only one proteome, representing orphan proteins, while 2649 clusters (32%) appeared in two or more proteomes but not in all, indicating accessory proteins. The remaining 2790 clusters (33%) were present across all proteomes, representing core proteins.

**Figure 3:**
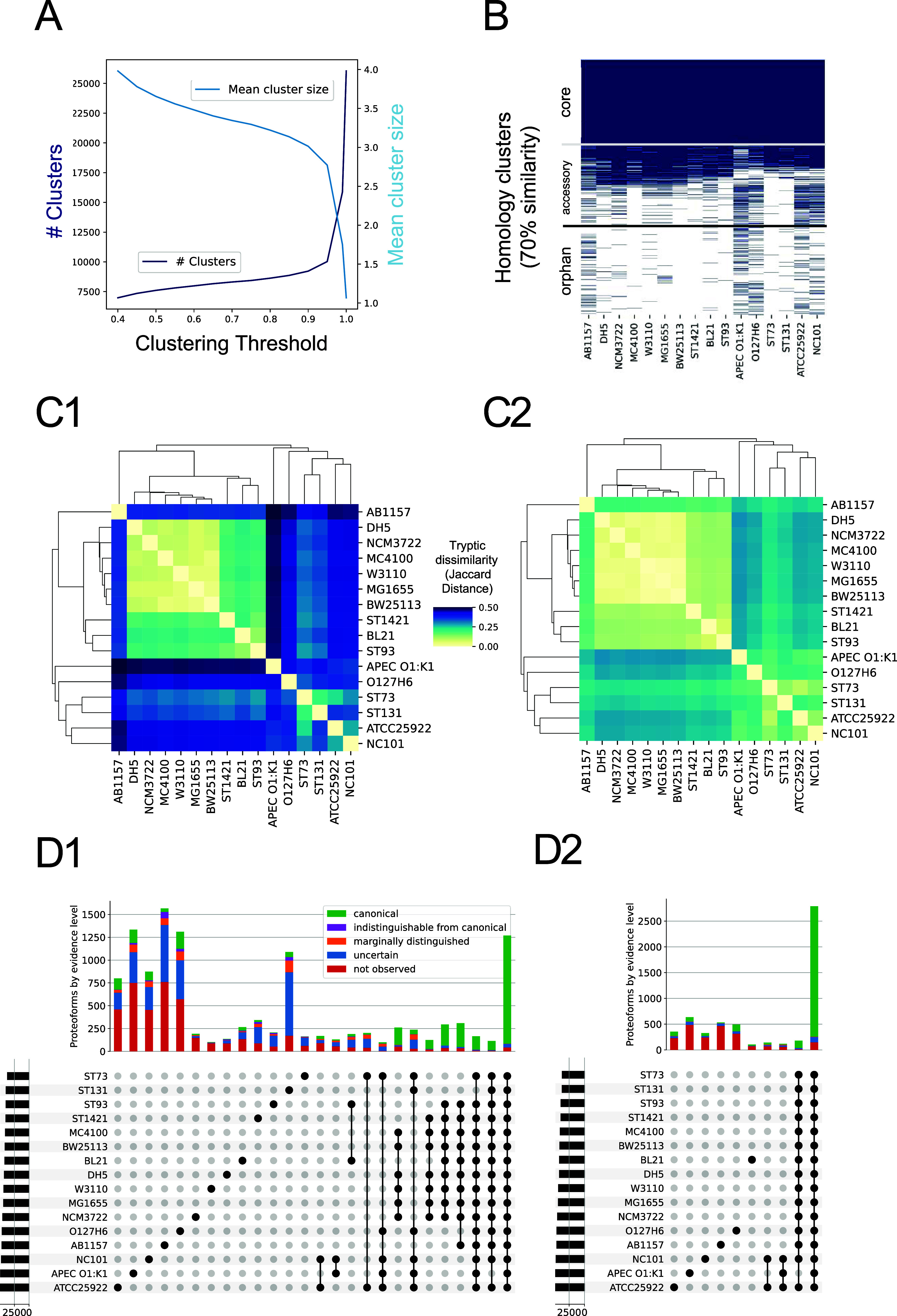
Predicted and observed *E. coli* pan-proteome. (A) Number of homology clusters and their sizes as a function of sequence similarity threshold used for clustering. (B) Homology clusters of *E. coli* pan-proteome after clustering at 70% sequence similarity. (C) Pairwise Jaccard distances of tryptic digests of UniProtKB proteomes (C1) vs pairwise Jaccard distances of tryptic digests of UniProtKB proteomes only considering peptides observed in the PeptideAtlas build (C2). (D) Upset plot of proteome clustering at 99% (D1) and 70% (D2) sequence similarity. Colors indicate highest level of evidence of all proteins in the cluster. Only sets with more than 100 protein clusters are shown.

At a 99% similarity threshold, the core proteome is significantly smaller than at 70%, with only 1269 proteins common across all strains (Supporting Figure S4). Only 247 proteins are shared with identical sequences across all strains.

#### Peptide Similarity and Observability in Proteomics Data Sets

The pairwise Jaccard distances of tryptic digests of UniProtKB proteomes (Figure 3C1) revealed distinct clustering patterns among *E. coli* strains. For example, the K-12 derivatives (MC4100, BW25113, DH5, W3110, MG1655, and NCM3722) are clustered together, while the proteomes from the pathogenic *E. coli* (e.g., NC101, APEC) show larger differences to all other strains.

When considering only the peptides observed in the PeptideAtlas build (Figure 3C2), the similarity between strains increased. Several factors likely contribute to this pattern. First, proteomic experiments generally capture only a fraction of the proteome, limiting experimental peptide coverage. Second, some sequence variations present in the strain proteomes may simply not have been represented in the analyzed samples. Third, even when such variants were present and corresponding spectra were acquired, search engines may have failed to assign a peptide sequence because of low spectral quality (for example, cofragmentation, mass inaccuracies, or missing fragment ions), or the resulting PSMs may have fallen below the applied FDR threshold.

#### Comparison of Proteome Clustering at High and Low Stringency

The proteome clustering at high (99%) and moderate (70%) sequence similarity thresholds exhibited distinct patterns (Figure 3D). The UpSet plot for 99% similarity (Figure 3D1) showed a greater number of unique protein clusters, indicative of strain-specific proteoforms. At 70% similarity (Figure 3D2), more clusters merged, emphasizing broader functional conservation. The color coding of protein clusters based on their level of evidence demonstrated that a significant fraction of the proteome has strong experimental support, while some clusters remained uncertain or unobserved.

The proteomes of strains ATCC 25922, APEC, NC101, AB1157, O127H6, and ST131 contain the highest numbers of unique proteoforms or proteins at 99% similarity (Figure 3D1). These numbers decrease significantly when the similarity threshold is slightly lowered, indicating that many of these proteoforms are from proteins shared across strains. However, some strain-specific clusters are retained even at lower thresholds (around 300–600 proteins, Figure 3D2), suggesting the presence of unique proteins for certain strains. While most of these strain-specific proteins lacked sufficient peptide evidence, a small subset was more confidently observed. These latter strain-specific proteins could potentially serve as markers for strain typing. For example, in the case of NC101, a pathogenic strain associated with adherent-invasive *E. coli* (AIEC) that currently lacks molecular markers,^74^ 56 strain-specific proteins were identified with canonical evidence, 23 of which were exclusively observed in NC101 strains. One such protein, cdiI, is part of the Contact-Dependent Inhibition (CDI) system, which is present in several *E. coli* strains, but the version in NC101 (Identifier UPI0001DBCD95/CDII_ECONC) differs significantly from the others. This protein was identified with 69.8% coverage and five unique peptides, all exclusively from NC101 samples. Given its strain specificity, its high detectability by mass spectrometry, and its lack of peptide matches in other strains, cdiI represents a promising candidate for strain typing.

The proteomes of pathogenic strains and isolates (ST73, NC101, APEC, ATCC25922) also show an overlap in proteins at 99% similarity. Their overlap could indicate conserved proteins necessary for virulence or survival in host environments. For the K-12 derivatives (MC4100, BW25113, DH5, W3110, MG1655, NCM3722), proteomes have a large overlap due to close genetic relationships.

Independent of the chosen similarity threshold, confident peptide evidence was observed for at least one protein in most of the core homology clusters. However, for a small fraction, no evidence was found. As these proteins are highly conserved across strains, they are probably important for basic functionalities and therefore present in the analyzed samples but missed due to technical limitations. In the following, we explore these limitations.

#### Dark Part of the Pan-Proteome Is Mostly Dark Due to Technical Limitations

For 6445 out of 27,806 (23%) proteoforms (or for 3276/8310 protein clusters at 70% similarity), no peptide evidence was observed in the *E. coli* PeptideAtlas. To investigate the effects of technical limitations and biases in the build, we characterized the observed and unobserved proteins by multiple characteristics: protein mass, length, number of tryptic peptides, cellular localization, and isoelectric point. To facilitate this analysis, we defined the observed pan-proteome as the set of proteoforms that have peptide evidence in the PeptideAtlas, independent of their tier.

Analysis of key properties revealed that a significant portion of the pan-proteome’s proteoforms is likely unobserved due to their chemical properties, which are not ideal for mass spectrometry analysis. Unobserved proteoforms predominantly have higher isoelectric points (Figure 4A), are mostly smaller, and give rise to less tryptic peptides that can be identified in mass spectrometry experiments (Figure 4B). Many of the unobserved proteoforms are also predicted to reside in the cytoplasmic membrane (Figure 4C), suggesting that they are primarily hydrophobic. This presents a challenge for detection, as hydrophobic proteins are typically less compatible with sample processing, protein isolation, and mass spectrometry due to their low solubility and low tryptic peptide yield.

**Figure 4:**
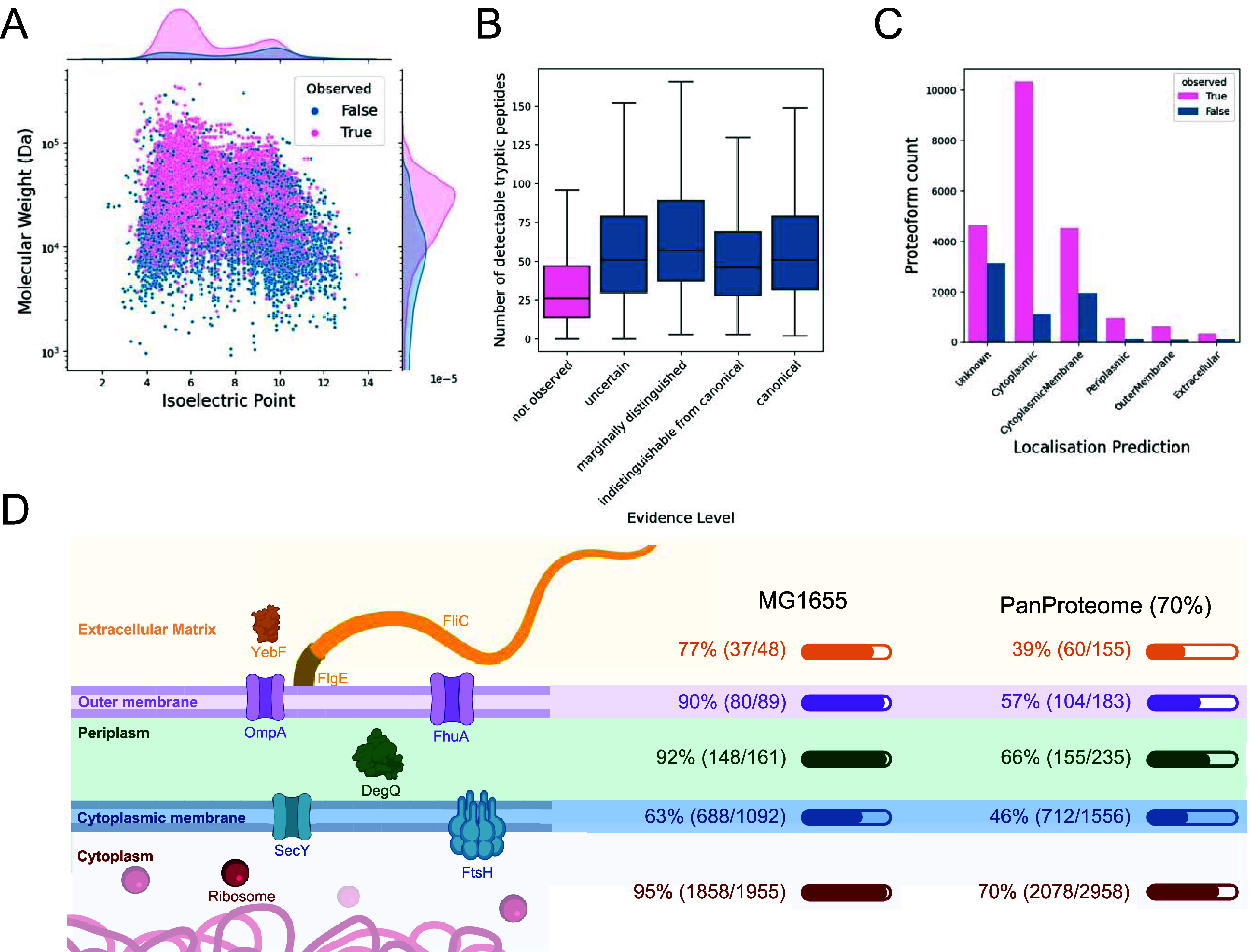
Properties of the observed vs the unobserved pan-proteome. (A) Distribution of proteoform weights and isoelectric points of observed vs unobserved proteoforms. (B) Distributions of number of detectable tryptic peptides across evidence tiers. (C) Number of observed vs unobserved proteoforms across cellular localizations as predicted by PSORTb. (D) Cellular locations of the proteoforms in *E. coli* cells. Left: Structure of the *E. coli* cell membrane system with example proteins. Right: Identification proportion with canonical evidence in the *E. coli* K-12 MG1655 proteome, and the pan-proteome clustered at 70% similarity. For 3224 homology clusters, and for 1059 MG1655 proteins, no localization could be predicted by PSORTb.

Despite these challenges, we were able to identify membrane proteins both in the cytoplasmic membrane and in the outer membrane (Figure 4D). This is likely thanks to the combination of fractionation and enrichment techniques and different sample preparation methods. For example, probe sonication has been shown to be effective for processing membrane proteins in bacterial systems.^41^ In addition, even proteins that are generally less amenable to mass spectrometry, such as highly hydrophobic or membrane-associated proteins, may still contain accessible domains or loop regions that yield one or more detectable peptides, facilitating their partial observation under optimal conditions.

### Post-Translational Modifications in *E. coli*

PTMs are essential regulatory mechanisms in all organisms, including bacteria, and they play a crucial role in the control of protein function, stability, localization, and interactions. In bacteria, PTMs can modulate a variety of processes such as metabolism, stress response, antibiotic resistance, and virulence. As highlighted by Macek et al.,^75^ PTMs in bacteria have gained significant attention due to their involvement in essential cellular processes, such as signal transduction, energy production, and response to environmental changes.

In our reprocessing of large-scale *E. coli* MS data, a wide variety of PTMs were detected. A total of 113,258 modification events were identified across the data sets, corresponding to multiple PTM types. These modification events (composed of protein residue and the modification type, e.g., Tyr23-phosphorylated) were categorized by their biological relevance, as per the Unimod classification.^76^ The most observed classes are *artifacts* (41,353 events), *isotopic labels* (32,005 events), *chemical derivatives* (21,628 events), and *post-translational modifications* (10,887 events) (Figure 5A). 830 modification events were assigned to more than one Unimod class, these are grouped under “Other”.

**Figure 5:**
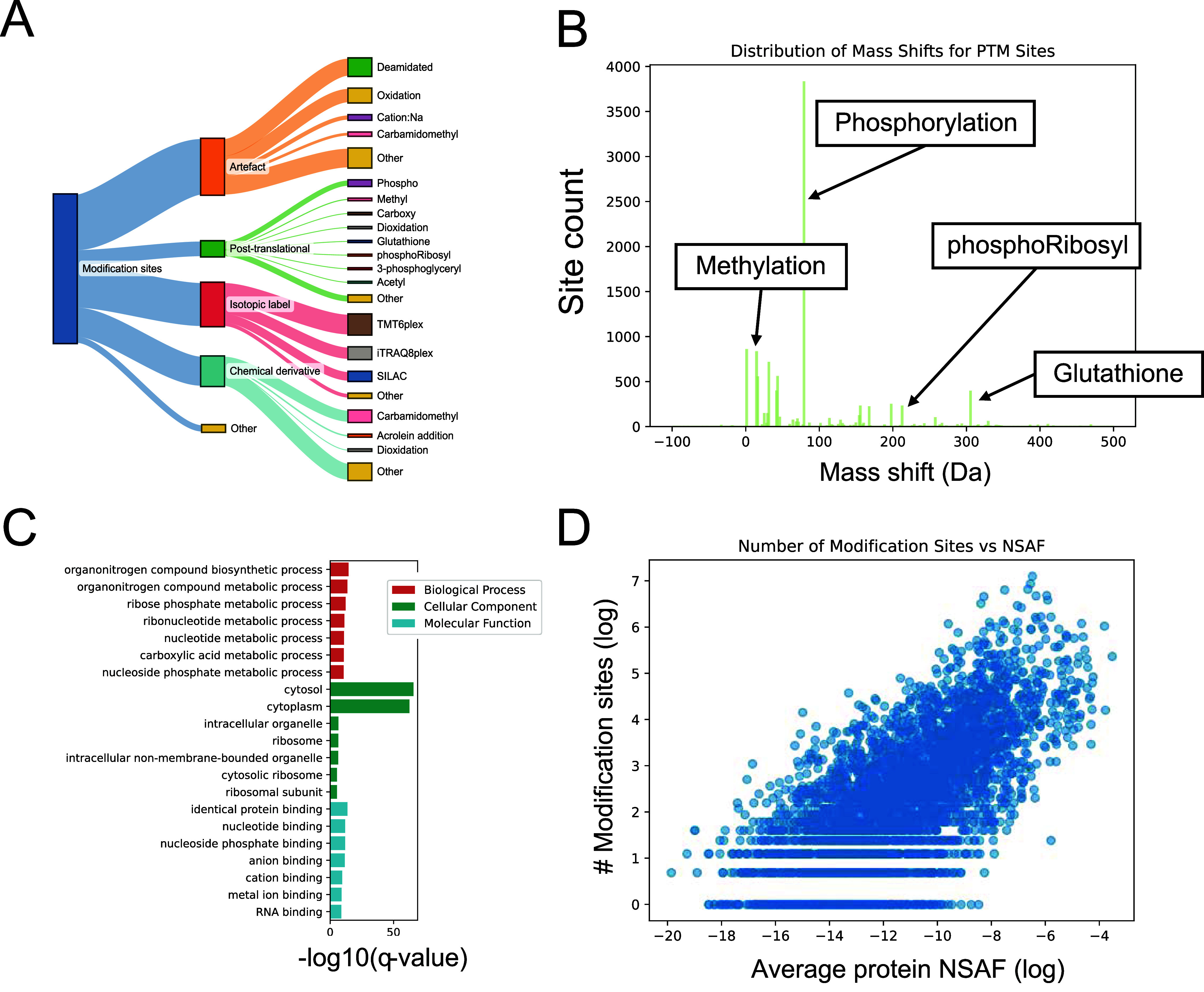
PTMs identified in the *E. coli* pan-proteome build. (A) Identified modifications and their classification according to Unimod. (B) Distribution of mass shifts induced by identified post-translational modifications, showing number of modification sites on *y*-axis. (C) Significantly enriched GO Terms of glutathionylated proteins. Colors indicate different GO Term classes. (D) Plotting protein abundance (NSAF) against number of modification sites shows that peptides from higher abundant proteins are more often identified in modified forms.

Artifacts comprise modifications that arise as byproducts of sample preparation, such as methionine oxidation resulting from exposure to atmospheric oxygen or reactive oxygen species during digestion and handling. The most prevalent artifactual modifications are deamidation (12,626 sites) and oxidation (9899 sites), primarily affecting asparagine and glutamine residues, and methionine residues, respectively. In contrast, chemical derivatives represent modifications introduced intentionally for experimental purposes, with carbamidomethylation being the most common (8115 sites), typically targeting cysteine residues during alkylation.

The selected projects for the build include four TMT, three SILAC, and one iTRAQ experiment, which is reflected in the number of identified isotopic labels. Other groups include glycosylations, nonclassified modifications, and co- or pretranslational modifications.

From a biological perspective, modification events classified as post-translational modifications are the most interesting. Here, the most prominent modification type is phosphorylation (3806 sites) due to the integration of phosphoenriched samples, followed by deamidation (854 sites), methylation (719 sites), and carboxylation (554 sites).

Among the identified PTMs, phosphorylation was found on the highest number of residues, with 3806 detected modification sites. Phosphorylation is one of the most studied and important PTMs in both eukaryotes and prokaryotes. In bacteria, phosphorylation plays a critical role in regulating signal transduction pathways, cell cycle progression, metabolism, and response to environmental changes. In *E. coli*, many key proteins involved in stress responses, signal transduction (e.g., two-component systems), and metabolic regulation are known to be phosphorylated. Phosphorylation often occurs on serine, threonine, and tyrosine residues. Our data indeed reveals a substantial presence of phosphorylation at serine (1753 sites), threonine (996 sites), tyrosine (553 sites), as well as histidine (371 sites). Other noncanonical amino acids were also reported as phosphorylated, although only at a small number of sites (C: 58, E: 38, D: 30, K: 4, R: 3). These might represent mislocalized phosphorylations or they could stem from false positive PSMs.

It has been previously described in the literature that phosphorylation sites are enriched in disordered regions of proteins in humans.^77,78^ In *E. coli*, we also observed more phosphorylated sites in disordered regions as compared to nonphosphorylated residues, however the difference was rather small (Supporting Figure S6).

Glutathione (GSH) is a critical antioxidant in bacterial cells, maintaining the redox balance by scavenging reactive oxygen species (ROS) and other free radicals. The modification of proteins by glutathione, referred to as glutathionylation, has been implicated in redox regulation, cellular protection against oxidative stress, and the regulation of key enzymes in *E. coli*. We identified 253 glutathione sites on cysteine residues. The identification evidence mainly derives from a project in which the bacteria were subjected to ciprofloxacin-induced antibiotic stress (PXD050358:4582/6185 glutathionylated PSMs). Metal ion binding proteins were significantly (adjusted p-value <0.05) overrepresented in glutathionylated proteins (Figure 5C), underlining the role of glutathionylation in cellular defense mechanisms, particularly in the homeostasis and resistance to metal ions.^79^

Phosphoribosylation, the modification of proteins by the addition of a phosphoribosyl group, is another important PTM in bacteria, particularly in the context of nucleotide metabolism and secondary metabolism. In our data, phosphoribosylation was found on glutamic acid (125 sites) and aspartic acid (94 sites), with some occurrences on arginine (7 sites). This modification is most likely involved in bacterial processes related to nucleotide biosynthesis and regulation, as phosphoribosylation is a critical step in purine and pyrimidine biosynthesis pathways. The presence of phosphoribosylation in *E. coli* may be linked to the regulation of metabolic pathways that are essential for cellular growth and proliferation, especially under varying environmental conditions.

To contextualize the PTMs identified in the PeptideAtlas build, we compared our results with the curated PTM annotations available in dbPTM.^80^ dbPTM lists 17,077 PTM sites for *E. coli*, of which 16,499 could be mapped to modification types considered in our analysis. Within the 113,258 PTM events identified in PeptideAtlas, 18,294 events corresponded to modification types curated by dbPTM. Of these, 836 modification events were found to overlap directly between the two resources.

We further investigated the potential bias of PTM site identifications. As modifications are thought to exist mostly at substoichiometric levels,^75^ it is likely that we will identify PTMs on proteins that are higher abundant to begin with. Indeed, the number of modification sites increases with protein abundance (Figure 5D).

We investigated whether the observed patterns of post-translational modifications could be explained by the available experimental metadata. Principal clustering of the runs was primarily driven by project affiliation, indicating pronounced batch effects as a major contributor to variation in PTM profiles (Supporting Figure S7A). Clustering by strain was also observed (Supporting Figure S7B); however, due to the strong correlation between strain and experimental batch, it remains challenging to disentangle the respective contributions of biological versus technical factors. No clear clustering was detected based on other metadata variables such as growth medium composition (Supporting Figure S7C,D), optical density, or other culture conditions. While this absence of signal does not exclude potential biological influence on PTMs, it is likely that such effects are obscured by dominant batch effects or limited by insufficient annotation coverage for certain variables.

## Discussion and Conclusion

In this study, we have presented comprehensive reprocessing and analysis of large-scale *E. coli* proteomics data from multiple global research projects. By employing both closed (TPP with MSFragger) and open (ionbot) search strategies, we have integrated data from 40 selected projects, resulting in the identification of 4755 high-confidence proteins. These identifications represent a significant contribution to the understanding of the *E. coli* proteome under a variety of conditions, including stress responses, antibiotic exposure, and genetic modifications. By using a core proteome derived from the well-annotated K-12 MG1655 strain and combining it with additional strains and phages, we have created a detailed *E. coli* PeptideAtlas, which serves as a valuable resource for future studies. It allows for the visualization and exploration of protein identifications across strains, with detailed annotations on coverage, abundance, and PTMs. Additionally, we detected evidence for previously unidentified proteins, providing strong experimental evidence for canonical proteins without protein evidence in UniProtKB.

To ensure compatibility with both search strategies, our analysis was restricted to data sets acquired using DDA, with the exception of one DIA data set, which was processed using only the TPP. This choice was motivated by the current limitations in open search capabilities for DIA data, as ionbot does not support this acquisition mode. Although DIA holds promise for improved reproducibility and depth of coverage, the inability to perform open modification searches on DIA data remains a constraint. We anticipate that as search engines evolve to accommodate open searches in DIA mode, future versions of this PeptideAtlas resource may be expanded to include such data sets, thereby further enhancing proteome coverage and biological insight. In addition, we expect the spectral libraries provided here to support future DIA studies of *E. coli* by extending existing TripleTOF-focused resources^4^ to Orbitrap-based acquisitions and by including libraries relevant to isobaric labeling workflows such as TMT and iTRAQ.

As we identified phage proteins in multiple runs, we suggest including bacteriophage proteins in the search space of database searches to improve completeness. Despite stringent efforts to maintain sterile conditions, bacteriophage contamination remains a major challenge. The inclusion of phage sequences in the database facilitates the detection of phage-related peptides, improving the spectrum identification rate and making it easier for researchers to account for and identify potential phage contamination and prophages in their studies. This approach is particularly valuable in settings where the likelihood of phage protein presence is elevated, such as laboratories that routinely handle bacteriophages, studies involving newly uncharacterized strains, or experiments that enrich for outer membrane vesicles. Moreover, incorporating these sequences can aid in improving false discovery rate (FDR) control by reducing the number of incorrectly assigned matches to bacterial proteins when spectra in fact originate from phage-derived peptides.

While significant progress has been made in making data publicly available, a key challenge remains in providing comprehensive annotation. In our study, a considerable amount of time was spent on manual data curation and annotation, as existing annotations were not standardized, incomplete, and sometimes even wrong. Improved and standardized metadata annotation by the original authors would greatly accelerate reprocessing efforts and facilitate the biological interpretation of the results.

To conclude, this work provides a high-quality, deeply annotated, and easily accessible resource that significantly enhances our understanding of the *E. coli* proteome across a wide range of biological contexts. By systematically reprocessing diverse data sets and integrating robust search strategies, we have built a PeptideAtlas that not only expands the coverage of known proteins, including previously unconfirmed ones, but also enables exploration of proteomic variation across strains and conditions. The PeptideAtlas build (called 2024–11 ionbot) is available online and includes all identified peptide-spectrum matches, post-translational modifications, and associated metadata. This comprehensive resource supports targeted mass spectrometry assay development, PTM-focused studies, and facilitates comparative analyses across strains. Ultimately, it represents a valuable foundation for future *E. coli* proteomics research, fostering data reuse, reproducibility, and discovery.

## Supplementary Material







## Data Availability

The PeptideAtlas build and spectral libraries are available online at https://peptideatlas.org/builds/ecoli/. Code for producing the figures and the search fastas are available on GitHub (https://github.com/CompOmics/ecoli-peptideatlas-manuscript) and Zenodo (10.5281/zenodo.17974410). The mass spectrometry proteomics data and associated metadata have been deposited to the ProteomeXchange Consortium via the PRIDE^81^ partner repository with the data set identifier PXD058808.

## Notes

Differences in experiment level spectrum matching rates between strains, projects, and mass spectrometer instruments (Figure S1); proteome sizes of included strains and phages (Figure S2); distribution of number of peptidoforms per homology cluster, as a function of similarity threshold (Figure S3); pan-proteome clustering with CD-HIT at 100% similarity (Figure S4); histogram of mass shifts induced by modifications (including all Unimod classes) identified in the build uniquely mapping to canonical proteins (Figure S5); distribution of ESMFold pLDDT values of all residues, STY residues, and phosphorylated residues (Figure S6); t-SNE of all runs according to post-translational modifications (Figure S7) (PDF)
Project_and_Run_Metadata: metadata on project level, experiment level, and run level (SDRF), modification mass shifts for the search settings, and proteome sizes (XLSX)
Modification_Sites.tsv: mass shifts, unimod names, classifications, and modified residues of the modification sites uniquely mapping to a canonical protein in the build; note that for the position in the protein, placeholder values were used for protein C- and N-terminal modifications (XLSX)
