# Supplementary material for: The Escherichia coli PeptideAtlas Build: Characterizing the Observed Escherichia coli Pan-Proteome and Its Post-Translational Modifications
Source: J Proteome Res. 2026 Jan 22;25(2):1027–41. doi: 10.1021/acs.jproteome.5c00902 (PMC12887991; doi:10.1021/acs.jproteome.5c00902)
Supplement: Supplementary file 1 [file pr5c00902_si_001.pdf]

Supporting information for publication

The *E. coli* PeptideAtlas Build: Characterizing the observed *Escherichia coli* pan-proteome and its post-translational modifications

Caroline Jachmann[1,2], Zhi Sun[3], Kevin Velghe[1,2], Florence Arsène-Ploetze[4], Aurélie Hirschler[5,6], Jasper Zuallaert[1,2], Christine Carapito[5,6], Robbin Bouwmeester[1,2], Kay Nieselt[7], Eric Deutsch[3], Lennart Martens[1,2,5,6]*, and Ralf Gabriels[1,2]# & Tim Van Den Bossche[1,2]#

# equal contributions

* corresponding author, Email: lennart.martens@ugent.be

1 CompOmics, VIB Center for Medical Biotechnology, VIB, 9052 Ghent, Belgium

2 Department of Biomolecular Medicine, Faculty of Medicine and Health Sciences, Ghent University, 9052 Ghent, Belgium

3 Institute for Systems Biology, Seattle WA 98109, USA

4 Institut de biologie moléculaire des plantes, CNRS, Université de Strasbourg, 67000 Strasbourg, France

5 BioOrganic Mass Spectrometry Laboratory (LSMBO), IPHC UMR7178, CNRS, Université de Strasbourg, 67200 Strasbourg, France

6 French Proteomics Infrastructure ProFI, UAR2048, 31077 Toulouse, France

7 Institute for Bioinformatics and Medical Informatics, University of Tübingen, 72076 Tübingen, Germany

Supplementary Figures

Suppl. Figure 1:
Differences in experiment level spectrum matching rates between strains, projects, and mass spectrometer instruments.

Suppl. Figure 2: Proteome sizes of included strains and phages.

Suppl. Figure 3:
Distribution of number of peptidoforms per homology cluster, as a function of similarity threshold.

Suppl. Figure 4: Pan-proteome clustering with CD-HIT at 100% similarity.

Suppl. Figure 5:
Histogram of mass shifts induced by modifications (including all Unimod classes) identi fied in the build uniquely mapping to canonical proteins.

Suppl. Figure 6: Distribution of ESMFold pLDDT values of all residues, STY residues, and phosphorylated residues.

Suppl. Figure 7: t-SNE of all runs according to post-translational modifications.

Supplementary Tables

Suppl. Table 1 (XLSX): Metadata on project level, experiment level, and run level (SDRF), modification mass shifts for the search settings, and proteome sizes.

Suppl. Table 2 (XLSX): List of modification sites.
Mass shifts, Unimod names, classifications,
and modified residues of the modification sites uniquely mapping to a canonical protein in the build.
Note that for the position in the protein, placeholder values were used for protein C- and N-terminal modifications.

Supplementary Figures

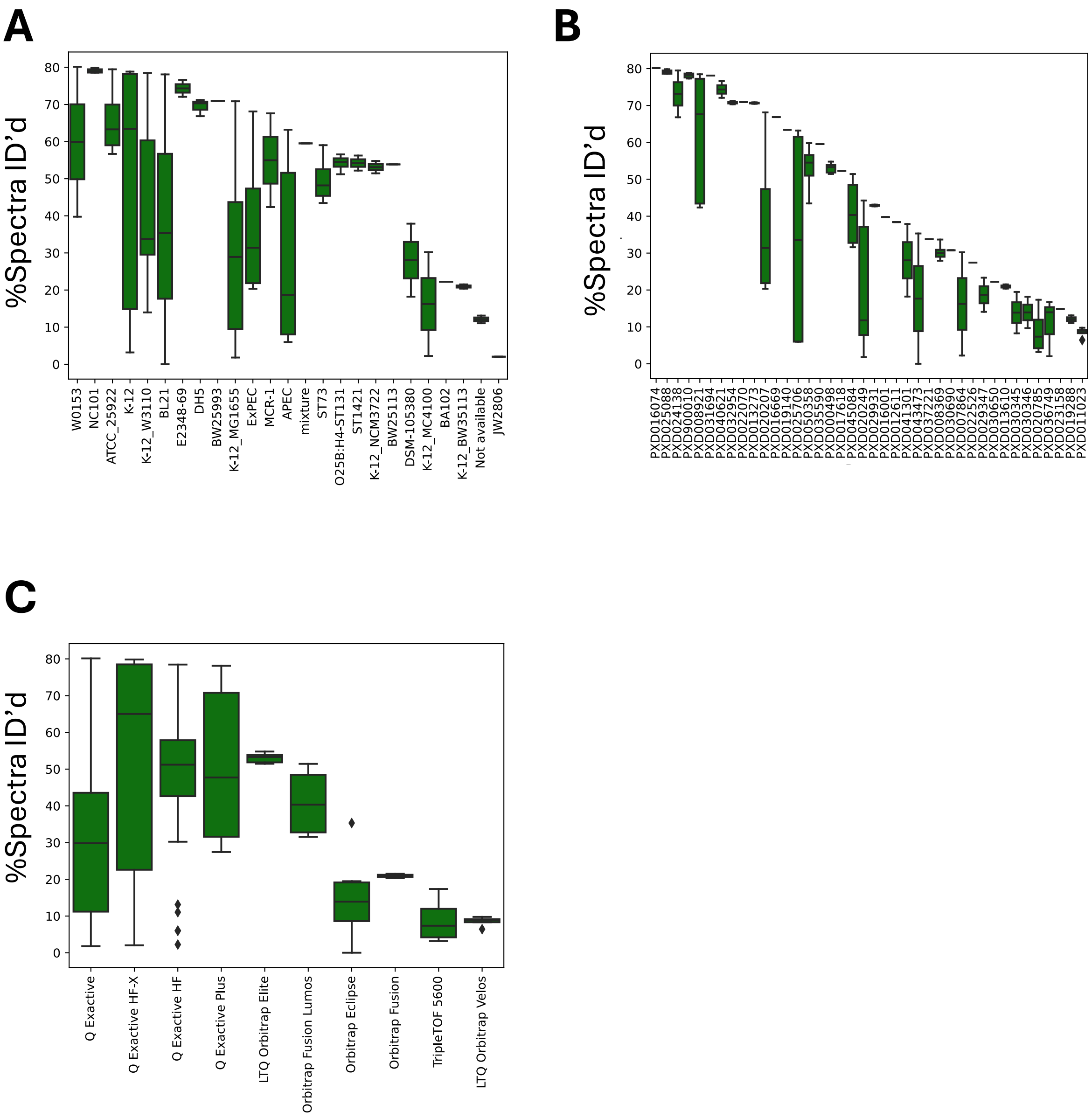

Supplementary Figure

1: Differences in experiment level spectrum matching rates between strains (**A**), project s (**B**), and mass spectrometer instruments (**C**).

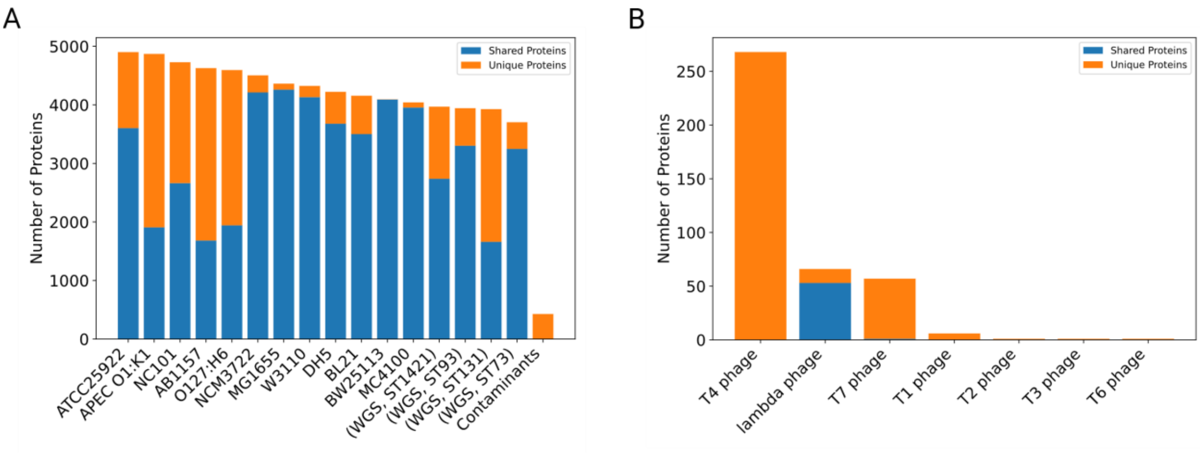

<u>Supplementary Figure 2:</u> Proteome sizes of included strains and phages.

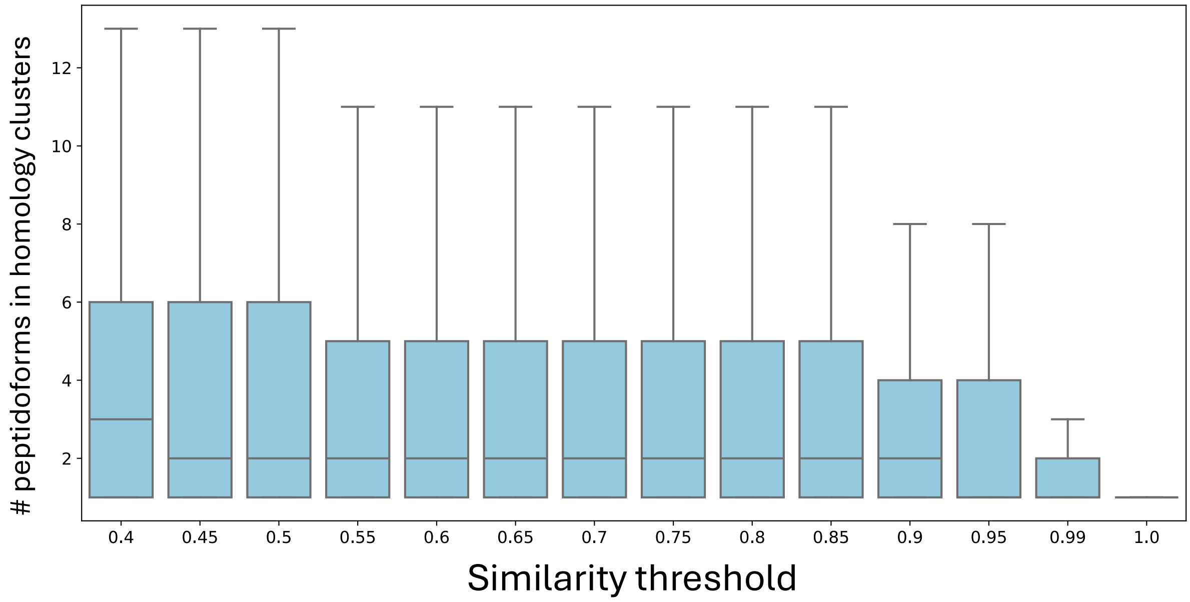

<u>Supplementary Figure 3:</u> Distribution of number of peptidoforms per homology cluster, as a function of similarity threshold.

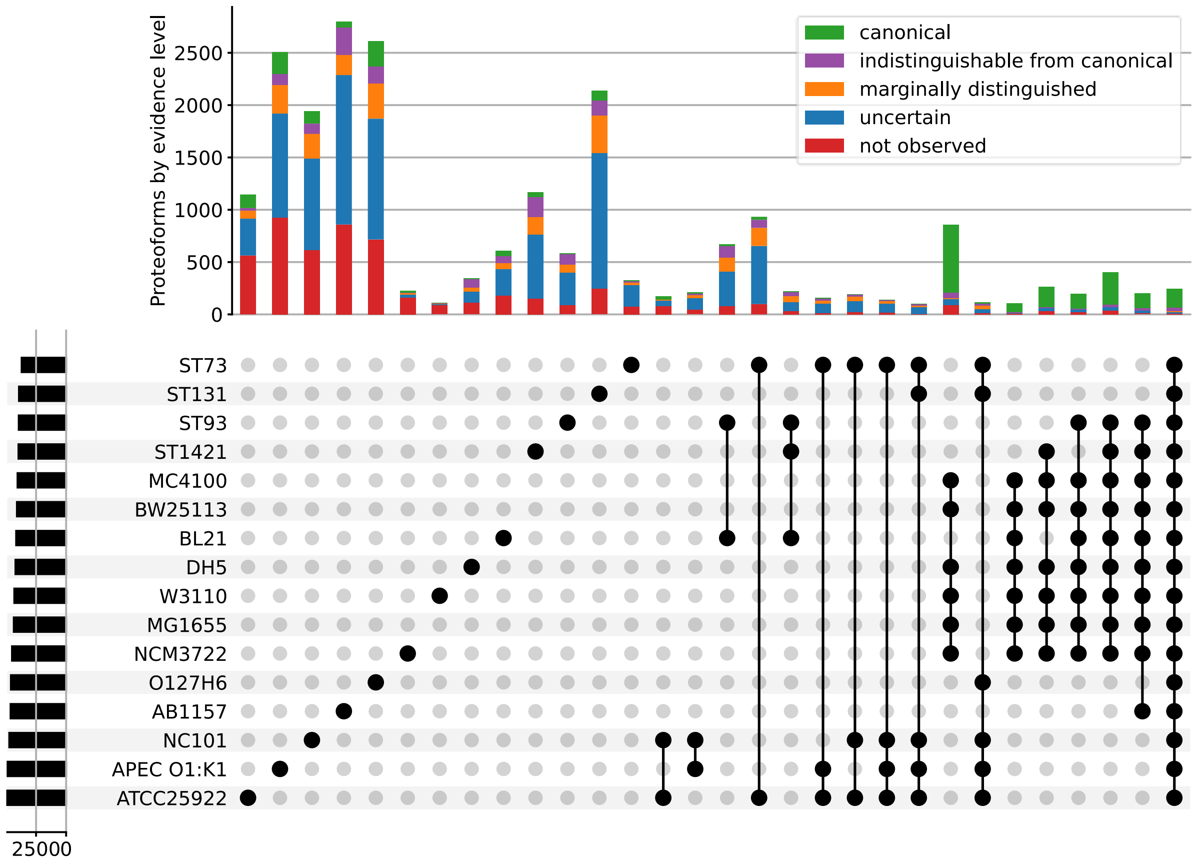

Supplementary Figure 4: Pan-proteome clustering with CD-HIT at 100% similarity.

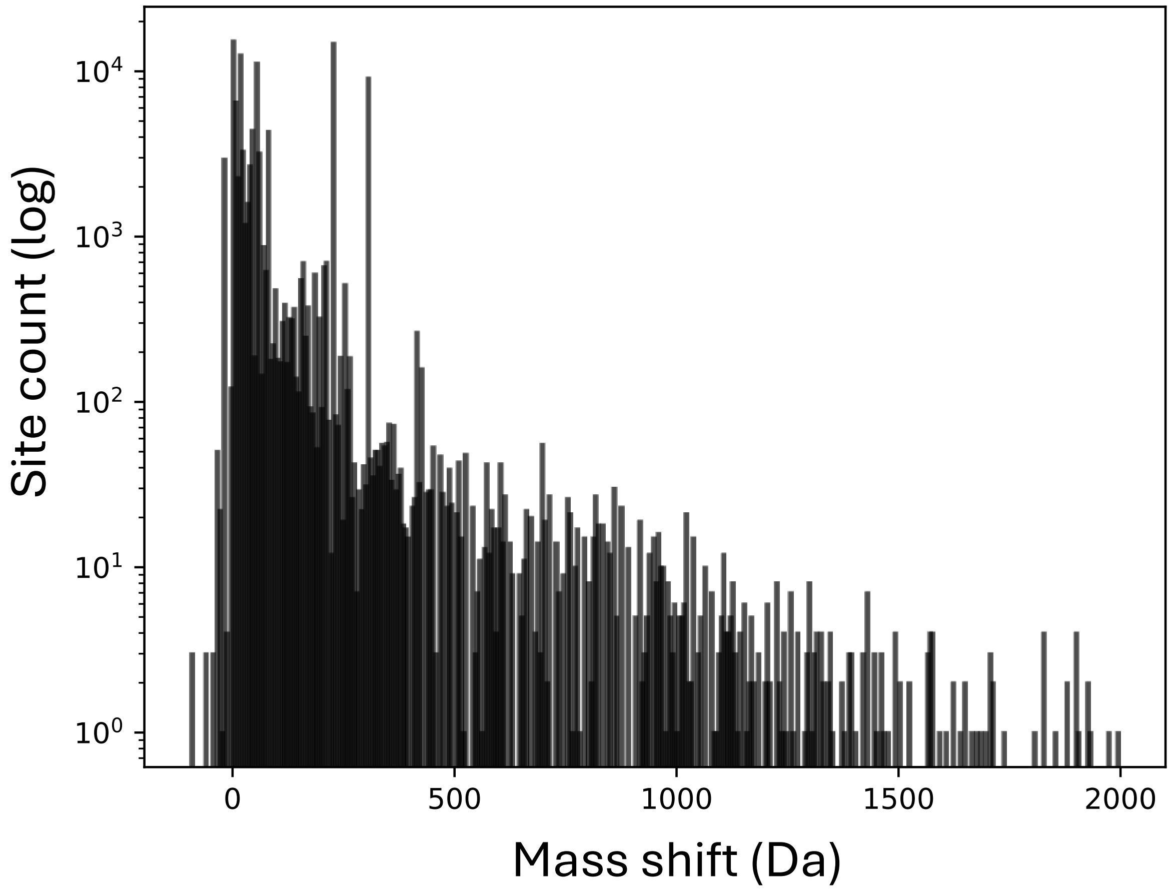

Supplementary Figure
5: Histogram of mass shifts induced by modifications (including all Unimod classes) ide ntified in the build uniquely mapping to canonical proteins.

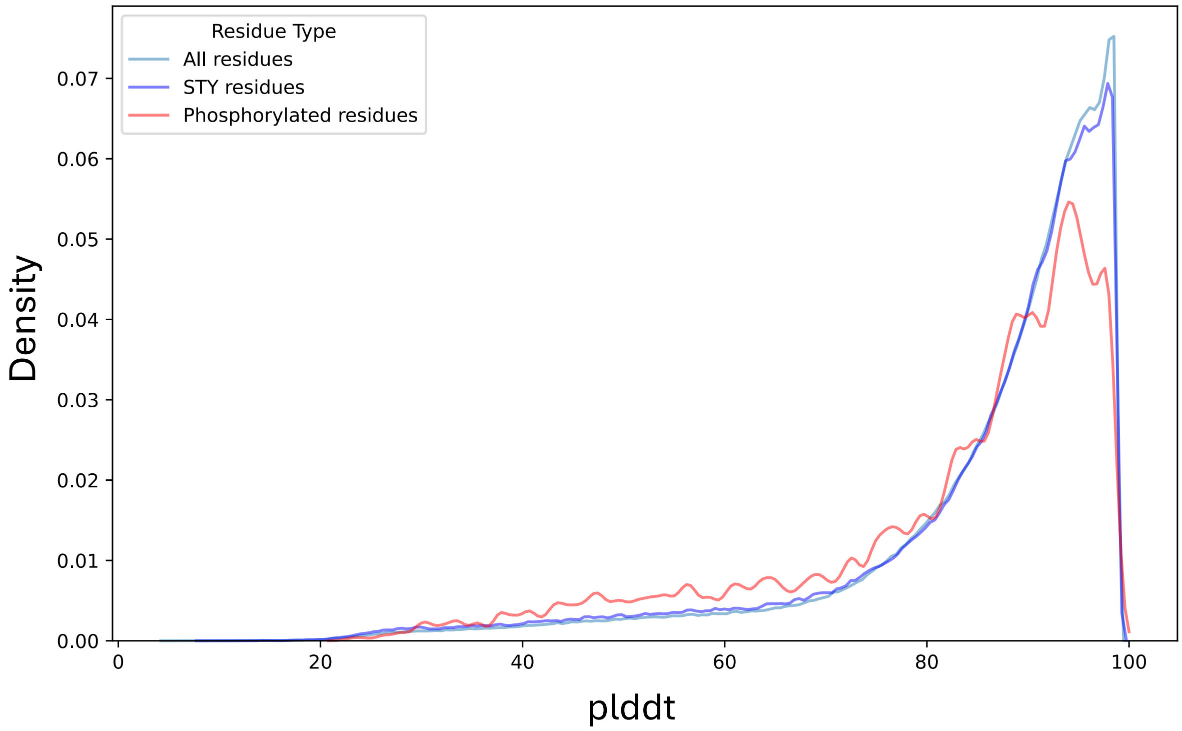

Supplementary Figure
6: Distribution of ESMFold pLDDT values of all residues (bright blue), STY residues (dark blue), and phosphorylated residues (red).
Data is based on ESMFold predictions of proteins in the highest confidence tier, and only phosphorylation sites matching uniquely to a canonical protein were included.

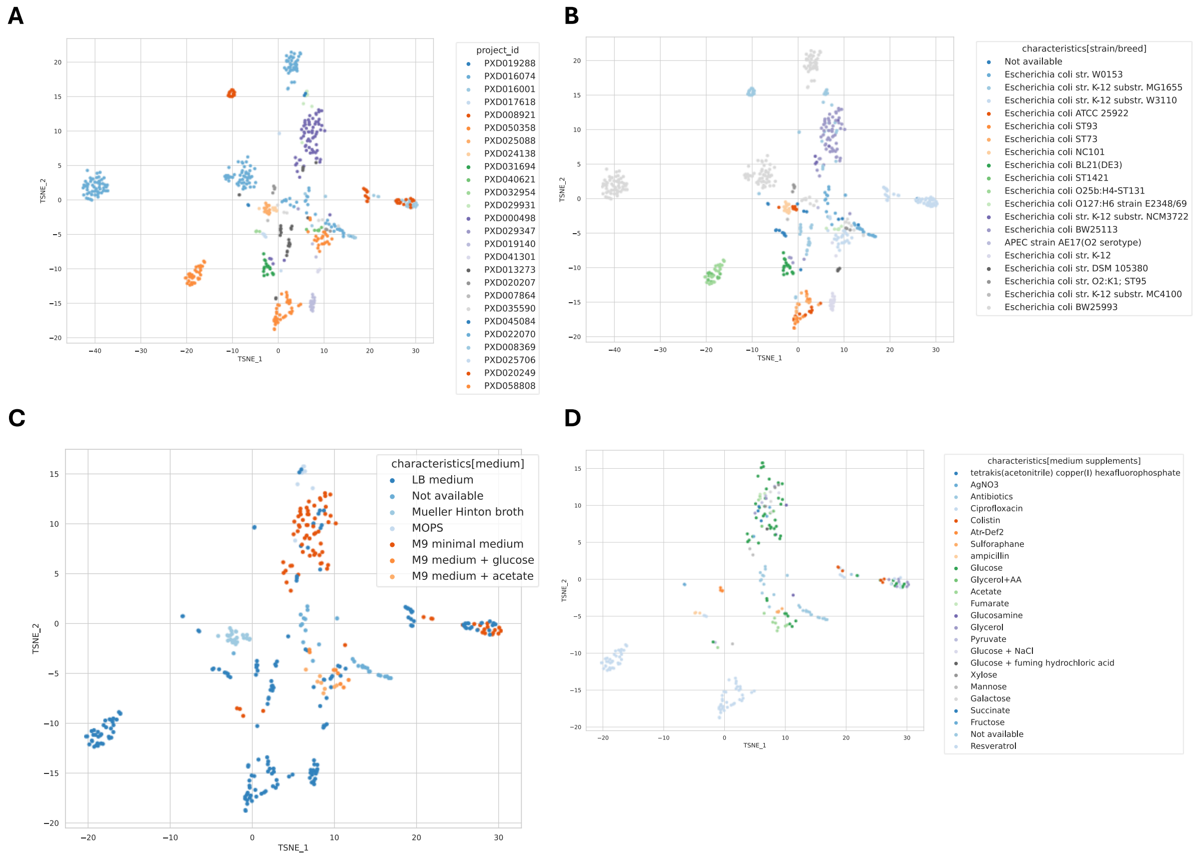

<u>Supplementary Figure 7:</u> t-SNE of all runs according to post-translational modifications, colored by experiment (**A**), strain (**B**), medium (**C**),and medium supplements (**D**). t-SNE based on the observation counts for each PTM per run, i.e. each run is represented by a vector specifying how many times each PTM was found in that run.

Note that runs without any observed modifications classified as post-translational are not represented.
